# The Proposed Molecular Mechanisms Used by Archaea for Fe(III) Reduction and Fe(II) Oxidation

**DOI:** 10.3389/fmicb.2021.690918

**Published:** 2021-07-01

**Authors:** Yiran Dong, Yawei Shan, Kemin Xia, Liang Shi

**Affiliations:** ^1^Department of Biological Sciences and Technology, School of Environmental Studies, China University of Geosciences, Wuhan, China; ^2^State Key Laboratory of Biogeology and Environmental Geology, China University of Geosciences, Wuhan, China

**Keywords:** dissimilatory Fe(III)-reducing archaea, dissimilatory Fe(II)-oxidizing archaea, redox proteins, electron transfer, molecular mechanisms

## Abstract

Iron (Fe) is the fourth most abundant element in the Earth’s crust where ferrous Fe [Fe(II)] and ferric Fe [Fe(III)] can be used by archaea for energy conservation. In these archaea-Fe interactions, Fe(III) serves as terminal electron acceptor for anaerobic respiration by a variety of archaea, while Fe(II) serves as electron donor and/or energy sources for archaeal growth. As no Fe is incorporated into the archaeal cells, these redox reactions are referred to as dissimilatory Fe(III) reduction and Fe(II) oxidation, respectively. Dissimilatory Fe(III)-reducing archaea (FeRA) and Fe(II)-oxidizing archaea (FeOA) are widespread on Earth where they play crucial roles in biogeochemical cycling of not only Fe, but also carbon and sulfur. To reduce extracellular Fe(III) (oxyhydr)oxides, some FeRA transfer electrons directly to the Fe(III) (oxyhydr)oxides most likely *via* multiheme *c*-type cytochromes (*c*-Cyts). These multiheme *c*-Cyts may form the pathways similar to those found in bacteria for transferring electrons from the quinone/quinol pool in the cytoplasmic membrane to the Fe(III) (oxyhydr)oxides external to the archaeal cells. Use of multiheme *c*-Cyts for extracellular Fe(III) reduction by both Domains of Archaea and Bacteria emphasizes an ancient mechanism of extracellular electron transfer, which is well conserved. Other FeRA, however, reduce Fe(III) (oxyhydr)oxides indirectly *via* electron shuttles. Similarly, it is proposed that FeOA use pathways to oxidize Fe(II) on the surface of the cytoplasmic membrane and then to transfer the released electrons across the cytoplasmic membrane inward to the O_2_ and NAD^+^ in the cytoplasm. In this review, we focus on the latest understandings of the molecular mechanisms used by FeRA and FeOA for Fe(III) reduction and Fe(II) oxidation, respectively.

## Introduction

By mass, iron (Fe) is the most abundant element on Earth and the fourth most abundant element in the Earth’s crust (Morgan and Anders, 1980). In the Earth’s biosphere, Fe exists mainly as metal ion of two different oxidation states: divalent ferrous Fe [Fe(II)] and trivalent ferric Fe [Fe(III)]. At the circumneutral pH and under oxic condition, Fe(II) is quickly oxidized to Fe(III) that is readily precipitated to solid Fe(III) (oxyhydr)oxides in the absence of chelator (Emerson et al., 2010). Thus, Fe(III) (oxyhydr)oxides are abundant in a variety of environments (e.g., soils, sediments and subsurface) where they can be used by microorganisms, including archaea, as the terminal electron acceptors for anaerobic respiration (Shi et al., 2016; White et al., 2016; Jiang et al., 2019). For example, the hyperthermophilic archaeon *Pyrobaculum islandicum* couples H_2_ oxidation to reduction of Fe(III) (oxyhydr)oxides for growth (Vargas et al., 1998). As Fe is not assimilated into microbial cells, microbial Fe(III) reduction for anaerobic respiration is referred to as dissimilatory Fe(III) reduction. Notably, microbial dissimilatory Fe(III) reduction is believed to be an ancient form of respiration (Vargas et al., 1998). In addition to H_2_, dissimilatory Fe(III)-reducing archaea (FeRA) also couple oxidation of methane (CH_4_), acetate and other organic compounds to Fe(III) reduction (Tor and Lovley, 2001; Kashefi et al., 2002; Beal et al., 2009). Moreover, FeRA are found in different environmental settings, ranging from the sediment of freshwater lakes to deep sea hyperthermal vents, where they are involved in biogeochemical cycling of not only Fe, but also carbon (C) (Slobodkina et al., 2009; Yamada et al., 2014; Weber et al., 2017).

In addition to oxygen (O_2_), the stability of Fe(II) in solution is also pH-dependent: the more acidic, the more stable (Emerson et al., 2010). Thus, Fe(II) can be oxidized by bacteria as well as archaea under anoxic condition or at acidic pH (Hafenbradl et al., 1996; Edwards et al., 2000; Valdes et al., 2008). For example, the hyperthermophilic archaeon *Ferroglobus placidus* oxidizes Fe(II) under anoxic condition and at circumneutral pH (Hafenbradl et al., 1996). The extremely acidophilic archaeon *Ferroplasma acidarmanus* oxidizes Fe(II) at pH 0–2.5 and the Fe concentration that is as high as 111 g/L (Edwards et al., 2000). Furthermore, the acidophilic dissimilatory Fe(II)-oxidizing archaea (FeOA) are prevalent in the environments of low pH, such as acid mine drainages (AMD) and acidic hot springs, where they play crucial roles in not only AMD formation, but also biogeochemical cycling of Fe, C as well as sulfur (S) (Edwards et al., 2000; Baker and Banfield, 2003; Johnson et al., 2012; Chen et al., 2016; Huang et al., 2016). Notably, the FeOA *Ferroplasma* spp. are the key members of microbial consortia used in biomining of copper (Cu) and gold (Au) that associate the minerals rich in Fe and S (Dopson et al., 2004; Hawkes et al., 2006).

The electron exchanges between microorganisms and the metal ions or others that are external to microbial cells are termed as microbial extracellular electron transfer (Shi et al., 2016; White et al., 2016; Jiang et al., 2019). FeRA can reduce extracellular Fe(III) (oxyhydr)oxides either directly or indirectly. For example, the hyperthermophilic archaeon *Geoglobus ahangari* reduces Fe(III) (oxyhydr)oxides most likely *via* its cell surface-exposed multiheme *c*-type cytochromes (*c*-Cyts) (Manzella et al., 2013, 2015). All the currently available data suggest that FeOA oxidize Fe(II) on the exterior side of the cytoplasmic membrane probably *via* their cell surface-exposed redox proteins, such as *b*-type cytochromes and the Cu-containing proteins (Kozubal et al., 2011; Castelle et al., 2015).

This review focuses on our current understandings of the molecular mechanisms underlying the ability of FeRA and FeOA to exchange electrons with Fe(III) and Fe(II), respectively.

## Iron (III) Reduction

### Dissimilatory Fe(III)-Reducing Archaea

#### Thermophilic and Hyperthermophilic Archaea

Thermophilic and hyperthermophilic archaea were among the first groups of archaea demonstrated conclusively to be capable of respiring on Fe(III) (Vargas et al., 1998; Slobodkin et al., 1999; Kashefi and Lovley, 2000; Kashefi et al., 2002, 2008). Among them, *Geoglobus acetivorans* and *Geoglobus ahangari* use only Fe(III) as the terminal electron acceptors, of which Fe(III) (oxyhydr)oxides are preferable (Kashefi et al., 2002; Slobodkina et al., 2009). Given that these thermophiles and hyperthermophiles are believed to be the most closely related to the last common ancestors of modern life, discovery of dissimilatory Fe(III) reduction by the thermophilic and hyperthermophilic archaea and bacteria provides microbiological evidences that dissimilatory Fe(III) reduction is an ancient form of microbial respiration (Liu et al., 1997; Pace, 1997; Vargas et al., 1998; Kashefi and Lovley, 2000). Consistent with these microbiological evidences are the geological evidences of abundance of H_2_ and Fe(III) (oxyhydr)oxides on pre-biotic Earth where microbial dissimilatory Fe(III) reduction occurred earlier than microbial reduction of sulfate (SO4^2–^), nitrate (NO_3_^–^) and O_2_ for respiration (Walker, 1984, 1987; Lovley, 1991).

#### Methanogens

Methanogens are groups of strictly anaerobic archaea that are directly involved in CH_4_ production (Thauer et al., 2008). Fe(III) can also be reduced for respiration by the methanogens that are phylogenetically and physiologically diverse (Vargas et al., 1998; Bond and Lovley, 2002; Bodegom et al., 2004; Liu et al., 2011a; Zhang et al., 2012, 2013; Yamada et al., 2014; Sivan et al., 2016; Prakash et al., 2019). For example, *Methanosarcina barkeri* couples H_2_ oxidation to reduction of Fe(III) (oxyhydr)oxides and Fe(III)-containing clay minerals, which lower the efficiency of CH_4_ formation by re-directing the electron originally for CH_4_ generation to Fe(III) reduction (Bond and Lovley, 2002; Bodegom et al., 2004; Liu et al., 2011a). Furthermore, the acetotrophic methanogen *Methanosarcina acetivorans* produces CH_4_ from the methyl group of acetate *via* fermentation whose *G*^*o*^′ = −36 kJ/mol (Deppenmeier and Muller, 2008; Prakash et al., 2019). Addition of ferrihydrite [Fe(III) (OH)_3_], however, increases CH_4_ production by *M. acetivorans* (Prakash et al., 2019). This increase of CH_4_ production is directly linked to the ferrihydrite reduction coupled to acetate oxidation whose *G*^*o*^′ = −707 kJ/mol (Roden and Lovley, 1993; Prakash et al., 2019). It is believed that a novel electron bifurcation mechanism facilitates the thermodynamically less favorable acetate fermentation to CH_4_ with the thermodynamically more favorable respiratory reduction of ferrihydrite (Prakash et al., 2019). This may result in increase of ATP level in the cytoplasm of *M. acetivorans*, which enhances production of methyl and carbonyl groups from acetate for methanogenesis (Yan and Ferry, 2018; Prakash et al., 2019).

#### Anaerobic Methane-Oxidizing Archaea

Phylogenetically related to methanogens, anaerobic methane-oxidizing archaea (ANME) can oxidize CH_4_ in conjunction with reduction of SO4^2–^, NO_3_^–^, Mn(IV) and Fe(III) (oxyhydr)oxides under anoxic condition, which play important roles in controlling CH_4_ emission on modern Earth (Boetius et al., 2000; Beal et al., 2009; Haroon et al., 2013; Timmers et al., 2017; Liang et al., 2019). On the early Earth without O_2_, this Mn(IV)- and Fe(III)-dependent anaerobic methane oxidation (AMO) is estimated to be capable of oxidizing nearly all available CH_4_ (Beal et al., 2009). ANME-1 and *Methanococcoides*/ANME-3 were implicated in CH_4_ oxidation that was coupled to reduction of Mn(IV) and Fe(III) (oxyhydr)oxides in a sample from the marine methane-seep sediment (Beal et al., 2009). Furthermore, some ANME-2 forms syntrophic relationship with sulfate-reducing bacteria (SRB) to mediate sulfate-dependent AMO *via* direct interspecies electron transfer (McGlynn et al., 2015). However, soluble molecules such as anthraquinone-2,6-disulphonate (AQDS), humic acids and Fe(III)-citrate, can serve as the terminal electron acceptors to substitute the roles of SRB in AMO by these ANME-2 (Scheller et al., 2016). These results also suggest that ANME-2 may use solid phase Mn(IV) and Fe(III) (oxyhydr)oxides as the terminal electron acceptors for anaerobic respiration (Scheller et al., 2016). Indeed, the ANME “*Candidatus* Methanoperedens ferrireducens,” “*Candidatus* Methanoperedens manganicus,” and “*Candidatus* Methanoperedens manganireducens” can catalyze AMO coupled to reduction of Mn(IV) and/or Fe(III) (oxyhydr)oxides (Ettwig et al., 2016; Cai et al., 2018; Leu et al., 2020a). Collectively, all these results clearly demonstrate the ability of some ANME to reduce Mn(IV) as well as Fe(III) (oxyhydr)oxides for anaerobic respiration. Notably, it is suggested that the syntrophic ANME were originally the free-living FeRA that explored their extracellular electron transfer capability to establish the direct electron transfer-dependent consortia with SRB (Scheller et al., 2016).

### The Proposed Molecular Mechanisms

**FIGURE 1:**
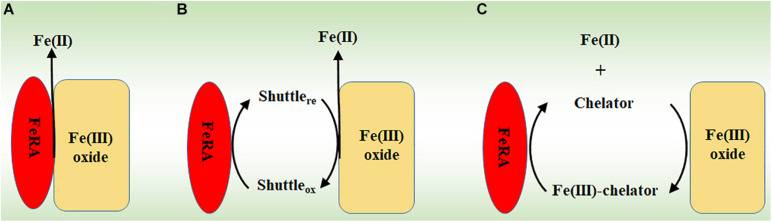
The mechanisms for reducing Fe(III) (oxyhydr)oxides by microorganisms. **(A)** Direct reduction *via* physical contact. **(B)** Indirect reduction *via* electron shuttles. **(C)** Indirect reduction *via* Fe(III) chelators. FeRA, Fe(III)-reducing archaea.

Microorganisms may transfer electrons to extracellular Fe(III) (oxyhydr)oxides either directly or indirectly. Direct reduction requires physical contact between the redox proteins on the microbial surfaces, such as multiheme *c*-Cyts, and the surfaces of Fe(III) (oxyhydr)oxides (Figure 1A; Xiong et al., 2006; Meitl et al., 2009). Indirect reduction may involve electron shuttles and Fe(III) chelators that are secreted by the microorganisms. The electron shuttles are first reduced by FeRA and the reduced shuttles then ferry the electrons to the Fe(III) (oxyhydr)oxides (Figure 1B; Marsili et al., 2008; von Canstein et al., 2008). Different from the electron shuttles, the Fe(III) chelators solubilize the Fe(III) from Fe(III) (oxyhydr)oxides and then bring the chelator-Fe(III) complexes back to the FeRA for reduction (Figure 1C; Gralnick and Newman, 2007; Marsili et al., 2008; Shi et al., 2009).

#### Direct Electron Transfer With Multiheme *c*-Cyts

##### Thermophilic FeRA

The thermophilic FeRA *G. ahangari* reduces Fe(III) (oxyhydr)oxides directly (Manzella et al., 2013). Although addition of AQDS stimulates the reduction, *G. ahangari* itself cannot reduce the Fe(III) (oxyhydr)oxides entrapped in the alginate beads of 12-kDa pore size, which is large enough for AQDS to pass through, but not for the archaeal cells (Manzella et al., 2013). Furthermore, compared to the fresh medium, addition of the supernatants of the stationary culture grown with Fe(III) (oxyhydr)oxides does not increase the reduction rate by *G. ahangari* (Manzella et al., 2013). Finally, addition of artificial chelators has no impact on the reduction of entrapped Fe(III) (oxyhydr)oxides by *G. ahangari* (Manzella et al., 2013). Thus, *G. ahangari* produces no electron shuttle or chelator for reducing Fe(III) (oxyhydr)oxides (Manzella et al., 2013). Similarly, neither *G. acetivorans* nor *F. placidus* is able to reduce the Fe(III) (oxyhydr)oxides inside the alginate beads (Mardanov et al., 2015; Smith et al., 2015). Thus, all these results show direct contact between Fe(III) (oxyhydr)oxides and *G. acetivorans*, *G. ahangari* and *F. placidus* is required for Fe(III) reduction (Manzella et al., 2013; Mardanov et al., 2015; Smith et al., 2015).

**FIGURE 2:**
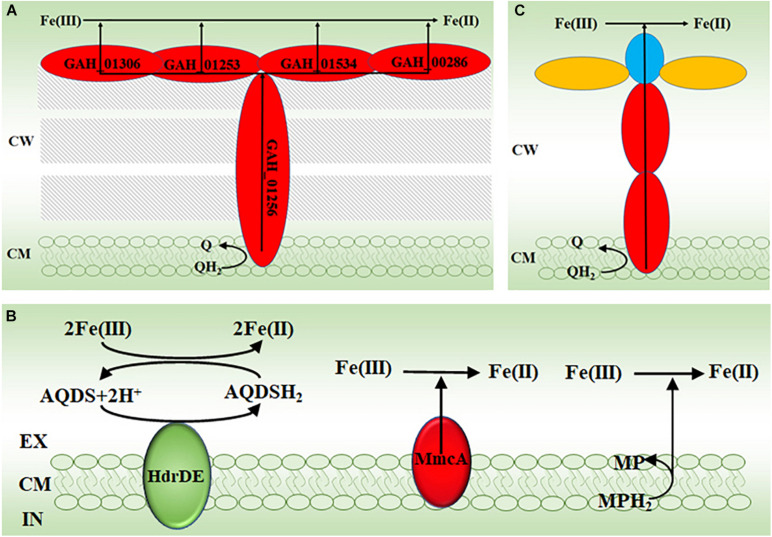
The proposed molecular mechanisms for reducing Fe(III) by archaea. **(A)** The proposed electron transfer pathway for extracellular reduction of Fe(III) by the thermophilic archaeon *Geoglobus ahangari*. **(B)** The proposed electron transfer mechanisms for extracellular reduction of Fe(III) by the methanogen *Methanosarcina acetivorans* (Yan et al., 2018). **(C)** The proposed electron transfer pathway for extracellular reduction of Fe(III) by the anaerobic methane-oxidizing archaea. EX, extracellular; CM, cytoplasmic membrane; CW, cell wall; IN, intracellular; AQDS, oxidized anthraquinone-2,6-disulphonate; AQDSH_2_, reduced AQDS; HdrDE, heterodisulfide reductase; MmcA, the cytoplasmic membrane multiheme *c*-type cytochrome A; MP, oxidized methanophenazine; MPH_2_, reduced MP; Q, quinone; QH_2_, quinol. The *c*-type cytochromes are labeled in red; the *c*-type cytochromes with S-layer protein domains are labeled in blue; the S-layer proteins are labeled in yellow.

Results from investigating bacterial extracellular electron transfer mechanisms demonstrate the importance of multiheme *c*-Cyts in direct reduction of Fe(III) (oxyhydr)oxides by bacteria (Shi et al., 2007, 2009, 2016; White et al., 2016; Jiang et al., 2019). Similarly, genomic sequencing results reveal that *G. ahangari* has 19 putative genes that encode multiheme *c*-Cyts and some of them are predicted to be on the archaeal cell surface (Manzella et al., 2015). Indeed, some heme-containing proteins can be mechanically sheared off from the cell surface of *G. ahangari* and this mechanic treatment renders the cells of *G. ahangari* unable to reduce Fe(III) (oxyhydr)oxides (Manzella et al., 2013). Moreover, transmission electron microscopy results show intimate association of the cells of *G. ahangari* with Fe(III) (oxyhydr)oxides (Manzella et al., 2013, 2015). Notably, *G. ahangari* possesses a putative decaheme *c*-Cyt of NapC/NirT family quinol dehydrogenase (GAH_01256) (Manzella et al., 2015). Investigation with the dissimilatory Fe(III)-reducing bacterium *Shewanella oneidensis* MR-1 reveals that tetraheme *c*-Cyt CymA, which is also a member of NapC/NirT family quinol dehydrogenase, is the key member of an electron transfer pathway for extracellular reduction of Fe(III) (oxyhydr)oxides. In this pathway, CymA oxidizes quinol in the cytoplasmic membrane and then transfers the released electrons to other *c*-Cyts that relay electrons eventually to the Fe(III) (oxyhydr)oxides on the bacterial cell surface (Shi et al., 2007, 2016; Marritt et al., 2012a,b; Mcmillan et al., 2012, 2013). Further analyses also suggest that the *c*-Cyts GAH_01306, GAH_00286 of GAH_01534, and GAH_01253 maybe the terminal reductases of *G. ahangari* for extracellular reduction of Fe(III) (Manzella et al., 2015). Although their proposed functions have not been experimentally verified, the *c*-Cyts GAH_01306, GAH_00286 of GAH_01534, GAH_01253 and GAH_01256 may function like those identified in *S. oneidensis* MR-1 to form an pathway for transferring electrons from the quinone/quinol pool in the cytoplasmic membrane, across the cell wall to the Fe(III) (oxyhydr)oxides on the cell surface of *G. ahangari* (Figure 2A).

Similar to *G. ahangari, G. acetivorans* and *F. placidus* also possess numerous genes for multiheme *c*-Cyts (Mardanov et al., 2015; Smith et al., 2015). Some of these *c*-Cyts of *G. acetivorans* contain the binding motifs for Fe(III) (oxyhydr)oxides, which were originally identified in the decaheme *c*-Cyts MtrC and OmcA. MtrC and OmcA are the terminal reductases used by *S. oneidensis* MR-1 for extracellular reduction of Fe(III) (oxyhydr)oxides (Xiong et al., 2006; Lower et al., 2008; Johs et al., 2010; Edwards et al., 2014, 2015, 2020; Mardanov et al., 2015). Furthermore, expressions of some of the *c*-Cyts with binding motifs for Fe(III) (oxyhydr)oxides are up-regulated when *G. acetivorans* is cultured under Fe(III)-reducing conditions and some of them are on the cell surface. Thus, *G. acetivorans* and *F. placidus* may reduce Fe(III) (oxyhydr)oxides *via* their cell surface-exposed multiheme *c*-Cyts (Mardanov et al., 2015; Smith et al., 2015). Given numerous *c*-Cyts found in these archaea, some of them may also form the electron transfer pathway similar to the proposed one of *G. ahangari* for extracellular reduction of Fe(III) (oxyhydr)oxides (Figure 2A; Mardanov et al., 2015).

##### Methanogen

The methyl-coenzyme M reductase (Mcr) is believed to catalyzes the key reaction of methane activation during anaerobic methane oxidation. The *mcr* genes are widespread in ANME. Addition of a *mcr* gene from an uncultivated ANME-1 enables the methanogen *M. acetivorans* to couple methane oxidation to Fe(III) reduction (Soo et al., 2016). Further characterizations show that the cytoplasmic membrane multiheme *c*-Cyt A (MmcA), heterodisulfide reductase (HdrDE), and methanophenazine (MP) in the cytoplasmic membrane are all capable of reducing the soluble Fe(III)-citrate either directly or indirectly (Figure 2B; Yan et al., 2018). Recent results suggested that MmcA was probably the terminal reductase for ferrihydrite (Prakash et al., 2019).

##### ANME

Multiheme *c*-Cyts are also believed to be crucial in direct electron transfer from ANME to SRB as well as in electron transfer from ANME to Mn(IV) and Fe(III) (oxyhydr)oxides (McGlynn et al., 2015; Wegener et al., 2015; Skennerton et al., 2017; Cai et al., 2018; Leu et al., 2020a,b). All these ANME genomes possess numerous multiheme *c*-Cyt-encoding genes. Some of these putative *c*-Cyts also contain S-layer domain, indicating that they are cell surface-exposed. Further chemical imaging analyses indeed detect cytochromes on these archaeal cell surface (McGlynn et al., 2015). Moreover, some of these multiheme *c*-Cyt-encoding genes are acquired by the ANME from the Fe(III)-reducing bacteria *via* lateral gene transfer (Skennerton et al., 2017; Leu et al., 2020b). Although their direct involvements in extracellular reduction of Fe(III) still remain to be demonstrated, the multiheme *c*-Cyts of these ANME may also form the pathways similar to those found in the bacteria and that proposed in other FeRA (Figure 2A) to transfer electrons from the quinone/quinol pool in the cytoplasmic membrane, across the cell wall to the Fe(III) (oxyhydr)oxides or the bacterial cells contacted on the archaeal cell surface (Figure 2C; McGlynn et al., 2015; Shi et al., 2016; Jiang et al., 2019; Leu et al., 2020b).

The crucial roles of multiheme *c*-Cyts in extracellular reduction of Fe(III) (oxyhydr)oxides and other extracellular substrates by both Domains of Archaea and Bacteria emphasizes that the multiheme *c*-Cyts-mediated pathway is an ancient and well conserved extracellular electron transfer mechanism.

#### Direct Electron Transfer With Other Redox Proteins

The hyperthermophilic FeRA *P. islandicum* reduces Fe(III) (oxyhydr)oxides and elemental sulfur [S(0)] directly as it is unable to reduce the Fe(III) (oxyhydr)oxides and S(0) inside the dialysis bags of 1.2–1.4 kDa pore size and produces no electron shuttle (Feinberg and Holden, 2006). It should be noted that *P. islandicum* lacks *c*-Cyt. Thus, *P. islandicum* must employ *c*-Cyt-independent mechanisms for direct reduction of Fe(III) (oxyhydr)oxides (Feinberg et al., 2008). Indeed, molybdopterin oxidoreductases of *Pyrodictium delaneyi* are suggested to transfer electrons directly to Fe(III) (oxyhydr)oxides (Kashyap and Holden, 2021).

#### Indirect Electron Transfer

*Pyrobaculum aerophilum* and *Pyrobaculum arsenaticum* reduce Fe(III) (oxyhydr)oxides indirectly as they can still reduce the Fe(III) (oxyhydr)oxides inside the dialysis bags of 1.2–1.4 kDa pore size that separate the archaeal cells and the (oxyhydr)oxides (Feinberg and Holden, 2006; Feinberg et al., 2008). Given that no chelated Fe(II) or Fe(III) is detected outside the bags, *P. aerophilum* is suggested to use electron shuttles to reduce the Fe(III) (oxyhydr)oxides inside the dialysis bags (Feinberg and Holden, 2006).

## Iron (II) Oxidation

### Dissimilatory Fe(II)-Oxidizing Archaea

#### The Neutrophilic FeOA

The FeRA *F. placidus* was originally isolated from a shallow submarine hydrothermal system at Vulcano, Italy as an Fe(II) oxidizer that could couple Fe(II) oxidation to NO_3_^–^ reduction with a pH optimum of 7 (Hafenbradl et al., 1996). Notably, *F. placidus* can fix CO_2_ and reduce NO_3_^–^ to N_2_O for chemolithoautotrophic growth when H_2_ is used as an electron donor (Vorholt et al., 1997). However, whether *F. placidus* can chemolithoautotrophically grow with Fe(II) and NO_3_^–^ still needs to be experimentally tested.

#### Acidophiles

Nearly all FeOA isolated so far are the acidophiles. They are abundant in the acidic and extremely acidic environments rich in Fe and S, such as acidic hot springs, AMD sites and bioleaching plants. These acidophilic FeOA are the key players in regulating biogeochemical cycling of C, Fe, and S, in AMD formation as well as in biomining of Cu, Au and other metals associated with the minerals that contain Fe and S (Baker and Banfield, 2003; Chen et al., 2016; Golyshina et al., 2016; Huang et al., 2016; Quatrini and Johnson, 2018). For example, *Metallosphaera yellowstonensis* was isolated from the Fe(III) oxide microbial mats in the acidic geothermal springs in Yellowstone National Park. It grows autotrophically with pyrite (FeS_2_) and Fe(II) sorbed on ferrihydrite at the optimal pH 2–3 and temperature 65–75°C (Kozubal et al., 2008). Notably, the Fe(III) oxide microbial mats also contain other microorganisms, such as Marsarchaeota and Geoarchaeota that reside under microaerobic condition and beneath the more oxic zone where *M. yellowstonensis* is abundant (Kozubal et al., 2012, 2013; Bernstein et al., 2013; Jay et al., 2018). Furthermore, the Fe(III)-reducing archaea are also found in these microbial mats (Kozubal et al., 2012). Thus, an active archaea-mediated Fe(II)/Fe(III) cycling is believed to occur in the mats (Kozubal et al., 2012). Moreover, *Metallosphaera sedula* was isolated from an acidic drain of a hot spring near Naples, Italy. It can grow chemolithoautotrophically on FeS_2_, sphalerite [(Zn,Fe)S], chalcopyrite (CuFeS_2_) and S(0) at pH 1–4.5 and temperature 50–80°C (Huber et al., 1989). Other *Metallosphaera* spp. that are isolated from the acidic hot springs of different locations can also grow by oxidizing Fe(II) and other metal ions (Kozubal et al., 2008, 2011; Liu et al., 2011b,c; Wheaton et al., 2019). Notably, *Metallosphaera* spp. belong to the order of *Sulfolobales*, among which *Sulfolobus metallicus, Sulfolobus tokodaii*, and *Acidianus brierleyi* (formerly known as *Sulfolobus brierleyi*) are all isolated from acidic hot springs and are all able to oxidize Fe(II) for growth (Brierley and Brierley, 1973; Segerer et al., 1986; Huber and Stetter, 1991; Suzuki et al., 2002; Bathe and Norris, 2007).

In addition to AMD sites, *Ferroplasma* spp. is also found in the bioleaching pilot plants globally. For example, *F. acidiphilum* was isolated from the bioreactor of a pilot plant at Tula, Russia for bioleaching the arsenopyrite (FeAsS) and FeS_2_ ores that contain Au. *F. acidiphilum* is an aerobe that oxidizes Fe(II), including FeS_2_, with an optimum pH of 1.7 for growth (Golyshina et al., 2000). Originally identified as *F. cupricumulans*, *Acidiplasma cupricumulans* was isolated from an industrial-scale CuFeS_2_ bioleach heap in Myanmar and it grew on Fe(II) at optimum pH 1–1.2 (Hawkes et al., 2006; Golyshina et al., 2009). It should be noted that all the tested *Ferroplasma* and *Acidiplasma* spp. are facultative anaerobes that can couple oxidation of yeast extract to Fe(III) reduction, which demonstrate that these *Ferroplasma* and *Acidiplasma* spp. are both FeOA and FeRA, depending on the ambient environmental conditions (Dopson et al., 2004; Zhou et al., 2008; Golyshina et al., 2009).

### The Proposed Molecular Mechanisms

**FIGURE 3:**
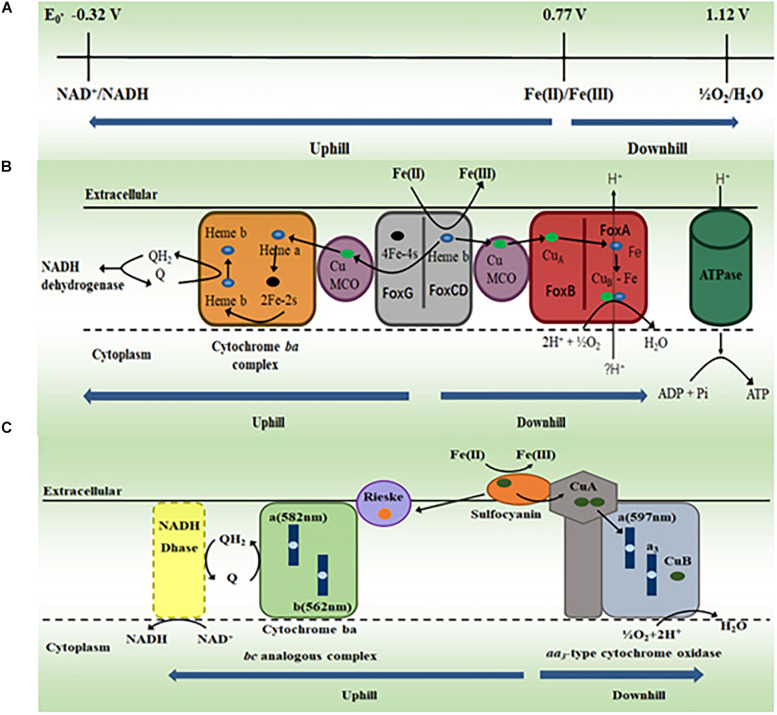
The proposed molecular mechanisms for oxidizing Fe(II) by acidophilic archaea. **(A)** Standard redox potentials (*E*_0_′) of NAD^+^/NADH couple at pH 6.5, Fe(II)/Fe(III) and O_2_/H_2_O couples at pH 2. Modified with permission from Bonnefoy and Holmes (2012)© (2012) Society for Applied Microbiology and John Wiley and Sons Ltd. **(B)** The proposed electron transfer pathway for oxidation of Fe(II) by *Metallosphaera yellowstonensis*. MCO, multicopper oxidase. Modified with permission from Kozubal et al. (2011)© (2011) American Society for Microbiology. **(C)** The proposed electron transfer pathway for oxidation of Fe(II) by *Ferroplasma acidiphilum.* Q, quinone; QH_2_, quinol. Modified with permission from Castelle et al. (2015)© (2015) Elsevier.

Fe(II) oxidation is a bioenergetic challenge for microorganisms, especially for autotrophic microorganisms. At pH 2, the redox potentials for Fe(II)/Fe(III) and O_2_/H_2_O couples are +0.77 V and +1.2 V, respectively. Thus, little energy can be derived from Fe(II) oxidation coupled to O_2_ reduction (Bird et al., 2011). Furthermore, the redox potential for NAD^+^/NADH couple is −0.32 V at pH 6.5, which is the pH found in the microbial cytoplasm. Thus, without any energy input, Fe(II) oxidation coupled to NAD^+^ reduction is thermodynamically unfeasible (Bonnefoy and Holmes, 2012). To overcome this thermodynamic barrier, the acidophilic FeOA use the energy from proton motive force to drive the electron transfer from Fe(II) oxidation to NAD^+^ reduction (i.e., uphill electron transfer). Although energy generation is limited, the electron transfer from Fe(II) oxidation to O_2_ reduction is thermodynamically feasible (i.e., downhill electron transfer) and thus can be catalyzed by microorganisms (Figure 3A; Bird et al., 2011; Bonnefoy and Holmes, 2012; Ilbert and Bonnefoy, 2013; Nitschke and Bonnefoy, 2016; Jiang et al., 2019).

#### Metallosphaera yellowstonensis

Sequencing results revealed that the genome of *M. yellowstonensis* contained a gene cluster that was also found in other FeOA, such as *M. sedula* and *S. metallicus*, in which this gene cluster was implicated in Fe(II) oxidation (Bathe and Norris, 2007; Auernik and Kelly, 2008; Kozubal et al., 2008). In this gene cluster, two of its protein products FoxCD are proposed to oxidize Fe(II) on the surface of the cytoplasmic membrane of *M. yellowstonensis* and transfer the released electrons in the cytoplasmic membrane to either O_2_ *via* the proposed downhill pathway or NAD^+^ *via* the proposed uphill pathway in the cytoplasmic membrane (Kozubal et al., 2011). The downhill electron transfer pathway may include multicopper oxidase (MCO) and terminal heme-copper oxidase FoxAB. The uphill electron transfer pathway may include MCO, Cytochrome *bc* complex, quinone/quinol pool and NADH dehydrogenase (Figure 3B; Kozubal et al., 2011). Given that they also have the *fox* gene cluster, the acidophilic FeOA *Acidianus copahuensis*, *M. sedula*, and *S. metallicus* are also suggested to oxidize Fe(II) *via* the mechanism similar to that of *M. yellowstonensis* (Bathe and Norris, 2007; Auernik and Kelly, 2008; Auernik et al., 2008; Bonnefoy and Holmes, 2012; Ilbert and Bonnefoy, 2013; Nitschke and Bonnefoy, 2016; Urbieta et al., 2017).

#### Ferroplasma acidiphilum

Sequencing results reveal no *fox* homolog in the genomes of *Ferroplasma* spp. (Tyson et al., 2004; Allen et al., 2007; Golyshina et al., 2017). Protein purification and characterization demonstrate that the blue copper protein sulfocyanin of *F. acidiphilum*, which is suggested to be on the exterior side of cytoplasmic membrane, oxidizes Fe(II) directly (Castelle et al., 2015). Sulfocyanin is part of an 850 kDa protein complex that also contains the *aa*_3_-type cytochrome oxidase. Thus, this cytoplasmic membrane protein complex is proposed to couple Fe(II) oxidation to O_2_ reduction (Figure 3C; Castelle et al., 2015). A 150 kDa protein complex is also isolated from the membrane fraction. This complex is believed to be the Rieske–cytochrome *b*-type complex that is suggested to mediate electron transfer from sulfocyanin to quinone in the cytoplasmic membrane (Figure 3C). The similar Fe(II) oxidation mechanism may also exist in other *Ferroplasma* spp. (Castelle et al., 2015). Thus, *F. acidiphilum* and most likely other *Ferroplasma* spp. oxidize Fe(II) *via* a mechanism different from that of *M. yellowstonensis.* Given that they are also different from those of bacteria (Bird et al., 2011; Bonnefoy and Holmes, 2012; Shi et al., 2012, 2016; Ilbert and Bonnefoy, 2013; Jiang et al., 2019), the molecular mechanisms for Fe(II) oxidation by FeOA must have evolved independently.

## Conclusion Remarks

The phylogenetically and physiologically diverse groups of FeRA and FeOA have been isolated from a variety of ecosystems worldwide. They are directly involved in biogeochemical cycling of Fe, Mn, C, and S. Furthermore, FeRA reduce Fe(III) (oxyhydr)oxides either directly or indirectly and multiheme *c*-Cyts are believed to be involved in the direct reduction of extracellular Fe(III) (oxyhydr)oxides most likely by forming the extracellular electron transfer pathways. Finally, FeOA are proposed to oxidize Fe(II) on the surface of the cytoplasmic membrane *via* the *b*-type cytochromes and Cu-containing proteins that maybe the part of the electron transfer pathways.

In bacteria, the extracellular electron transfer pathways connect extracellular redox transformation of Fe with intracellular metabolic activities. These pathways consist of redox and structural proteins as well as redox molecules (Table 1) [for reviews, see Bird et al. (2011), Bonnefoy and Holmes (2012), Ilbert and Bonnefoy (2013), Shi et al. (2016), White et al. (2016), Jiang et al. (2019)]. Some of these pathways are rigorously characterized (Shi et al., 2016; White et al., 2016; Jiang et al., 2019). In addition to the extracellular electron transfer pathways, extracellular extensions, such as *Geobacter* conductive pili (G-pili) and multiheme *c*-Cyt-based nanowires, have also been identified and characterized for transferring electrons to the Fe(III) (oxyhydr)oxides distant from bacterial surfaces (Table 1; Filman et al., 2019; Lovley and Walker, 2019; Reguera and Kashefi, 2019; Wang et al., 2019; Yalcin et al., 2020).

**TABLE 1:** Comparison of different types of proteins and redox molecules that are or are suggested to be involved in Fe(III) reduction or Fe(II) oxidation by bacteria and archaea.

Cellular locations	Bacteria		Archaea	
		
	Fe(III) reduction	Fe(II) oxidation	Fe(III) reduction	Fe(II) oxidation
Outer membranes or cell wall	Porin-cytochrome complex, *c*-Cyt^1^, G-pili^2^, *c*-Cyt-based nanowires, flavins.	Porin-cytochrome complex, *c*-Cyt.	*c*-Cyt, molybdopterin oxidoreductases	
Periplasms	*c*-Cyt, flavin-binding protein, flavins.	*c*-Cyt, iron-sulfur protein, copper-containing protein.		
Cytoplasmic membranes	*c*-Cyt, flavin-binding protein, quinone/quinol pool.	*c*-Cyt, NADH DHase^3^, photoreaction center, *aa*_3_ oxidase, *bc*_1_ complex, *cbb*_3_ oxidase, quinone/quinol pool.	*c*-Cyt, MP^4^, HdrDE^5^, unidentified redox molecules, quinone/quinol pool.	*b-*Cyt^6^, copper-containing protein, NADH DHase, *aa*_3_ cytochrome oxidase, heme-copper oxidase, *bc*_1_ complex, *ba* complex, quinone/quinol pool.
References*	Shi et al., 2016; White et al., 2016; Lovley, 2017a,b; Light et al., 2018; Filman et al., 2019; Jiang et al., 2019; Light et al., 2019; Lovley and Walker, 2019; Reguera and Kashefi, 2019; Wang et al., 2019; Edwards et al., 2020; Yalcin et al., 2020	Bird et al., 2011; Bonnefoy and Holmes, 2012; Ilbert and Bonnefoy, 2013; Nitschke and Bonnefoy, 2016; Shi et al., 2016; Jiang et al., 2019	Manzella et al., 2015; Mardanov et al., 2015; McGlynn et al., 2015; Smith et al., 2015; Yan et al., 2018; Jiang et al., 2019; Prakash et al., 2019; Leu et al., 2020b; Kashyap and Holden, 2021	Auernik and Kelly, 2008; Kozubal et al., 2011; Bonnefoy and Holmes, 2012; Ilbert and Bonnefoy, 2013; Castelle et al., 2015; Nitschke and Bonnefoy, 2016

^1^*c*-Type cytochrome.^2^*Geobacter* conductive pili.^3^NADH dehydrogenase.^4^Methanophenazine.^5^Heterodisulfide reductase.^6^*b*-Type cytochrome.*Some of the references are recent reviews.

Similar to those found in bacteria, multiheme *c*-Cyts and quinone/quinol pool are suggested to be the key components of the electron transfer pathways involved in extracellular reduction of Fe(III) by archaea. Likewise, Cu-containing proteins, NADH dehydrogenases, *aa*_3_ oxidase, *bc*_1_ complex, and quinone/quinol pool are implicated as the key components of the electron transfer pathways for Fe(II) oxidation by both bacteria and archaea (Table 1). Thus, archaea and bacteria may share these redox proteins and molecules for Fe(III) reduction and/or Fe(II) oxidation. However, identification of the redox proteins and molecules unique for archaeal Fe(III) reduction, such as molybdopterin oxidoreductases, HdrDE and MP, and those unique for bacterial Fe(III) reduction (e.g., flavins, flavin-binding proteins, G-pili and *c*-Cyt-based nanowires) demonstrate the diverse mechanisms that have evolved independently among different groups of archaea and bacteria for exchanging electrons with Fe(III) and Fe(II) (Table 1).

Compared to those in bacteria, the molecular mechanisms for reduction of Fe(III) (oxyhydr)oxides and oxidation of Fe(II) by archaea have been much less characterized and remain speculative, which is mainly attributed to the lack of genetically tractable model FeRA and FeOA. Thus, development of the representative model systems is crucial for further understanding these electron transfer mechanisms at molecular level. Currently, *M. acetivorans* and *M. barkeri* are the two genetically tractable methanogens for investigating molecular mechanisms for Fe(III) reduction. Although it is demonstrated to reduce soluble Fe(III), whether MmcA of *M. acetivorans* can reduce Fe(III) (oxyhydr)oxides remains uncharacterized. Furthermore, a novel *c*-Cyt-independent extracellular electron transfer mechanism has recently been identified and characterized in Gram-positive bacteria (Light et al., 2018, 2019). Whether the similar mechanism also exists in archaea remains uninvestigated. Thus, future research should focus on bridging these knowledge gaps.

Identification and characterization of archaeal electron transfer mechanisms at molecular level will not only substantially advance our understanding of how microorganisms exchange electrons with Fe(III) and Fe(II), but also lay solid foundations for further developments of FeRA- and FeOA-based biotechnology. For example, molecular understanding of electron transfer mechanisms from archaeal cell surface to their cytoplasmic membranes will certainly help develop electromethanogenesis *via* the synthetic biology approach (Cheng et al., 2009).

## Author Contributions

All authors listed have made a substantial, direct and intellectual contribution to the work, and approved it for publication.

## Conflict of Interest

The authors declare that the research was conducted in the absence of any commercial or financial relationships that could be construed as a potential conflict of interest.
